# Sulourea-coordinated Pd nanocubes for NIR-responsive photothermal/H_2_S therapy of cancer

**DOI:** 10.1186/s12951-021-01042-9

**Published:** 2021-10-14

**Authors:** Xiaoyang Guo, Jia Liu, Lingdong Jiang, Wanjun Gong, Huixia Wu, Qianjun He

**Affiliations:** 1 The Education Ministry Key Laboratory of Resource Chemistry, Joint International Research Laboratory of Resource Chemistry of Ministry of Education, Shanghai Key Laboratory of Rare Earth Functional Materials, and Shanghai Municipal Education Committee Key Laboratory of Molecular Imaging Probes and Sensors, College of Chemistry and Materials Science, Shanghai Normal University, Shanghai, 200234 China; 2 Central Laboratory, Longgang District People’s Hospital of Shenzhen & The Third Affiliated Hospital (Provisional) of The Chinese University of Hong Kong, Shenzhen, 518172 Guangdong China; 3 Guangdong Provincial Key Laboratory of Biomedical Measurements and Ultrasound Imaging, National-Regional Key Technology Engineering Laboratory for Medical Ultrasound, School of Biomedical Engineering, Health Science Center, Shenzhen University, Shenzhen, 518060 Guangdong China

**Keywords:** Pd nanocubes, Hydrogen sulfide, Photothermal therapy, Gas therapy, Nanomedicine

## Abstract

**Background:**

Photothermal therapy (PTT) frequently cause thermal resistance in tumor cells by inducing the heat shock response, limiting its therapeutic effect. Hydrogen sulfide (H_2_S) with appropriate concentration can reverse the Warburg effect in cancer cells. The combination of PTT with H_2_S gas therapy is expected to achieve synergistic tumor treatment.

**Methods:**

Here, sulourea (Su) is developed as a thermosensitive/hydrolysable H_2_S donor to be loaded into Pd nanocubes through in-depth coordination for construction of the Pd-Su nanomedicine for the first time to achieve photo-controlled H_2_S release, realizing the effective combination of photothermal therapy and H_2_S gas therapy.

**Results:**

The Pd-Su nanomedicine shows a high Su loading capacity (85 mg g^−1^), a high near-infrared (NIR) photothermal conversion efficiency (69.4%), and NIR-controlled H_2_S release by the photothermal-triggered hydrolysis of Su. The combination of photothermal heating and H_2_S produces a strong synergetic effect by H_2_S-induced inhibition of heat shock response, thereby effectively inhibiting tumor growth. Moreover, high intratumoral accumulation of the Pd-Su nanomedicine after intravenous injection also enables photothermal/photoacoustic dual-mode imaging-guided tumor treatment.

**Conclusions:**

The proposed NIR-responsive heat/H_2_S release strategy provides a new approach for effective cancer therapy.

**Graphic abstract:**

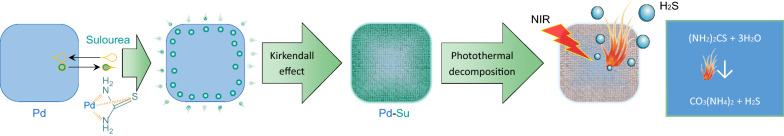

**Supplementary Information:**

The online version contains supplementary material available at 10.1186/s12951-021-01042-9.

## Background

In recent years, gas therapy (GT) has attracted much attention because of its unique therapeutic effects on some diseases, including inflammation, cardiovascular diseases, cancer, *etc.* [1–6]. Hydrogen sulfide (H_2_S), a colorless gas with the odor of rotten eggs, is considered to be the third gasotransmitter in mammals following the nitric oxide and carbon monoxide [7, 8]. As a kind of endogenous gasotransmitter, H_2_S exhibits significant physiological/pathological regulation functions. It has been indicated that H_2_S is involved in the regulation of the nervous system, cardiovascular system, and immune system in the human body [9, 10]. H_2_S also plays an important role in the development and treatment of tumors. Low level of endogenous H_2_S can promote tumor growth, while appropriate high concentration of H_2_S can exert a tumor suppressing effect [11, 12]. H_2_S can maintain the homeostasis of biological energy and reverse the Warburg effect in cancer cells, thereby blocking their survival pathways while protecting normal cells from damage [2, 13]. In order to achieve effective cancer therapy using H_2_S, it is necessary to transport the H_2_S donor to the tumor site with a suitable carrier and then use exogenous or endogenous stimuli to control the release of H_2_S in the tumor [14]. Sulourea (SC(NH_2_)_2_, Su) is a low-toxicity compound that can release H_2_S by hydrolysis and heat treatment, so it may serve as a thermosensitive H_2_S donor [15, 16].Therefore, we here hypothesize that photothermal agents can be used to load Su for near-infrared (NIR)-responsive co-release of heating and H_2_S to realize efficient gasothermal combined treatment of tumor.

Photothermal therapy (PTT) utilizes exogenous NIR irradiation to trigger the intratumoral photothermal transducers to locally generate hyperthermia for destroying tumors [17–19]. In recent years, a variety of photothermal agents have been developed for tumor treatment [20–22]. Among them, palladium (Pd) nanomaterials have received much attention due to high NIR photothermal conversion efficiencies, tunable size, and good biocompatibility. Up to now, Pd nanomaterials with varied morphologies have been designed to serve as PTT and photoacoustic imaging (PAI) agents for cancer treatment and monitoring [23–28]. Although PTT is featured with non-invasiveness, high efficiency, and low side effects, the generated hyperthermia usually results in the up-regulation of the heat shock protein expression, thereby increasing the heat stress tolerance of cancer cells and reducing the therapeutic outcome of PTT [29]. Therefore, it would be satisfactory to rationally combine PTT with other cancer treatment strategies and overcome the limitations of single PTT treatment through synergistic treatment. In view of the attractive merits of H_2_S-based GT, H_2_S is considered as an ideal candidate that can combine with PTT to achieve better anti-cancer effect [30].

**Scheme 1.**
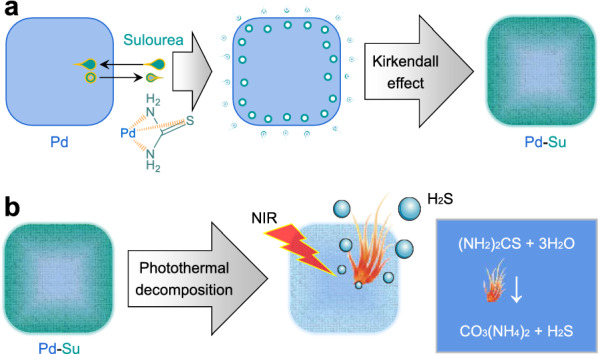
Schematic illustration of the synthesis of Pd-Su nanomedicine (**a**), and the NIR-responsive release of heating and H_2_S from Pd-Su nanomedicine (**b**)

In this work, a nanoplatform based on the coordination-mediated loading of Su within Pd nanocubes has been conveniently constructed for on-demand H_2_S release and effective photothermal/gas combination therapy (Scheme 1). Su can not only be firmly attached on Pd via the strong coordination, but also even penetrate into Pd nanocubes by the Kirkendal effect (Scheme 1a), achieving a high Su loading capacity. The resulting Pd-Su nanomedicine possesses the following advanced features: (1) significant enhancement of NIR absorption and photothermal conversion efficiency compared to Pd nanocubes; (2) high responsiveness and controllability for NIR-photothermal responsive release of H_2_S; (3) high accumulation in the tumor site after intravenous injection; (4) excellent photothermal/photoacoustic dual-mode imaging to guide the tumor treatment. Therefore, such an ingenious therapeutic nano-platform offers a new conceptual strategy for NIR-responsive photothermal/H_2_S therapy of cancer.

## Results and discussion

### Synthesis and characterization of Pd nanocubes and Pd-Su nanomedicine

**Fig. 1:**
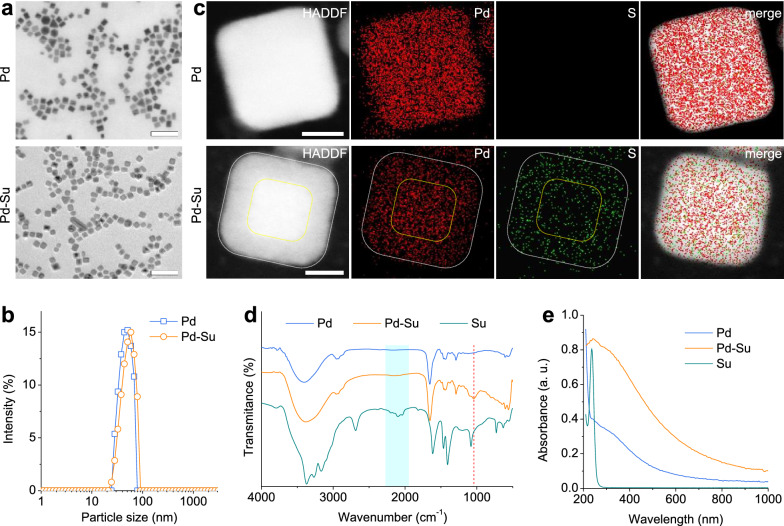
Characterization of Pd nanocubes and Pd-Su nanomedicine. **a** TEM images of Pd nanocubes and Pd-Su nanomedicine (scale bars, 100 nm). **b** DLS data of Pd nanocubes and Pd-Su nanomedicine. **c** EDS elemental mappings of Pd nanocubes and Pd-Su nanomedicine (scale bars, 5 nm). **d** FTIR spectra of Pd nanocubes, Pd-Su nanomedicine, and Su. **e** UV–Vis-NIR spectra of Pd nanocubes, Pd-Su nanomedicine, and Su (20 μg mL^−1^)

Pd nanocubes were synthesized according to the method reported in the literature, [23] using sodium tetrachloropalladate(II) (Na_2_PdCl_4_) as the precursor, ascorbic acid as the reducing agent, polyvinyl pyrrolidone (PVP) as the capping agent, and KBr as the inducer. KBr can induce the preferential growth of {100} crystal planes in Pd nanocubes, which was attributed to the selective and strong adsorption of Br^–^ ions on Pd {100} crystal planes [31]. Then, Su was loaded into Pd nanocubes to synthesize the Pd-Su nanomedicine via the coordination of thione sulfur and amino nitrogen with the surface Pd atoms [32–34]. The transmission electron microscopy (TEM) and the scanning electron microscopy (SEM) images (Fig. 1a and Additional file 1: Figure S1a) showed that the as-prepared Pd nanocubes exhibited uniform morphology and good dispersion, with a mean edge length of 9.1 ± 1.7 nm, and their hydrated particle size was measured to be 34.0 ± 9.8 nm by dynamic light scattering (DLS) (Fig. 1b). After Su loading, the resulting Pd-Su nanomedicine still kept the uniform nanocube morphology (Additional file 1: Figure S1b, S2), but the hydrated particle size was slightly increased (43.0 ± 14.3 nm), possibly owing to the massive Su modification/coordination on the surface of Pd nanocubes.

To confirm the successful loading of Su, energy dispersive X-ray spectroscopy (EDS) element mapping of Pd-Su nanomedicine and Pd nanocubes were analyzed and compared (Fig. 1c). As expected, no S element was detected in the Pd nanocubes, while the Pd and S elements were clearly found in the Pd-Su nanomedicine. More importantly, Su penetrated in the side of Pd nanocrystal to form a kind of core−shell structure by stealing Pd atoms from outside to inside by strong coordination, where higher amount of Su distributed in the shell (Fig. 1c), owing to the Kirkendal effect. This is the main reason why the hydrated particle size of Pd-Su nanomedicine increased slightly after Su loading. It resulted in high Su loading capacity of 85 mg/g, which was determined by thermal gravimetric analysis (TGA) (Additional file 1: Figure S3). Furthermore, the attenuated total reflectance Fourier transform infrared (ATR-FTIR) spectra showed the shift of the characteristic stretching band of C=S bond at about 1081 cm^-1^ toward lower wavenumber (Fig. 1d), suggesting the successful coordination between Su and Pd. [35] The X-ray diffraction (XRD) pattern (Additional file 1: Figure S4) indicated that Pd nanocubes have a typical cubic crystal structure (PDF#46-1043). The Pd-Su nanomedicine exhibited a XRD pattern similar to that of Pd nanocubes, indicating that the interface coordination for Su loading did not change the crystal structure of Pd nanocubes (Additional file 1: Figure S5). But the crystalline of Pd slightly decreased after Su loading, owing to in-depth etching of Su.

### Photothermal effect

The ultraviolet-visible-NIR (UV-Vis-NIR) absorption spectra of Pd nanocubes, Pd-Su nanomedicine, and Su are shown in Fig. 1e. Free Su showed no absorption in the wavelength range of 300~1000 nm, while both Pd nanocubes and Pd-Su nanomedicine exhibited significant absorption in the entire wavelength range. Moreover, due to the coordination effect of Su on the surface of Pd nanocubes, the absorption of Pd-Su nanomedicine in the NIR region was significantly greater than that of Pd nanocubes under the same particle concentration, which is beneficial to improve the NIR photothermal effect of Pd-Su nanomedicine.

**Fig. 2:**
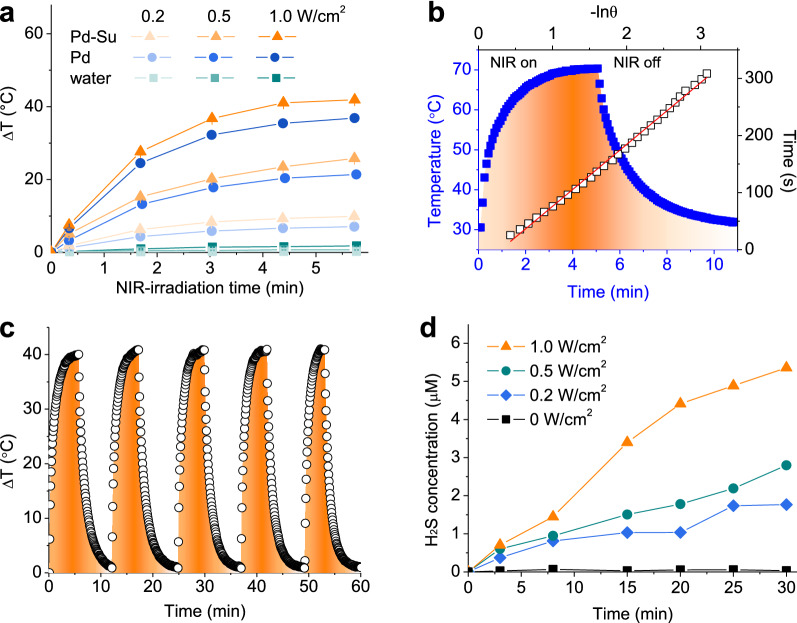
NIR-photothermal effect of the Pd-Su nanomedicine and NIR-responsive H_2_S release from the Pd-Su nanomedicine. **a** photothermal curves of Pd nanocubes and Pd-Su nanomedicine in water (200 μg mL^−1^) at different laser power densities. **b** Photothermal heating and cooling process of the Pd-Su solution (2 mg mL^−1^) under 808-nm laser irradiation (0.5 W cm^−2^) which was turned off after irradiation for 5 min, together with the plot of cooling time versus negative natural logarithm of the temperature driving force obtained from the cooling stage. **c** Recycling heating profiles of the Pd-Su nanomedicine for evaluating the photothermal stability. **d** Monitoring H_2_S release from the Pd-Su nanomedicine (4 mg mL^−1^) under the irradiation of 808-nm laser with different power densities (0.0, 0.2, 0.5, and 1.0 W cm^−2^) at different time points (0, 3, 8, 15, 20, 25, and 30 min) by the HSN-2 probe

Under the 808-nm laser irradiation, the photothermal temperature increase of Pd nanocubes and Pd-Su nanomedicine at the same concentration (200 μg mL^-1^) was measured using a thermal imaging camera. As indicated in Fig. 2a, the temperature of the material solutions increased with the increase of power density, with the maximum temperature elevation of 36.9 °C and 41.9 °C for Pd nanocubes and Pd-Su nanomedicine at 1.0 W cm^-2^, respectively. In contrast, deionized water only had a weak temperature elevation (2 °C) even at the highest power density (1.0 W cm^-2^). Compared to Pd nanocubes, Pd-Su nanomedicine showed much higher temperature rise under the same conditions. Even at a moderate power density of 0.5 W cm^-2^, the temperature rise of Pd-Su solution still reached ~25.8 °C. The photothermal conversion efficiency (*η*) of Pd nanocubes at 808 nm was calculated to be 53.4% based on the maximum steady-state temperature (Additional file 1: Figure S6), which is consistent with the literature reports [23, 36]. By comparison, the Pd-Su nanomedicine showed a much higher photothermal conversion efficiency (69.4%) at 808 nm (Fig. 2b), possibly due to the coordination between Su and Pd. These results indicated that the Pd-Su nanomedicine exhibited much better photothermal performance than Pd nanocubes. The photothermal stability of the Pd-Su nanomedicine was also evaluated (Fig. 2c). As the laser was switched on and off repeatedly for five times, the maximum temperature rise did not change significantly, indicating that the Pd-Su nanomedicine had good photothermal stability. Therefore, the Pd-Su nanomedicine could serve as an ideal photothermal therapeutic agent to provide heat for stimulating Su hydrolysis to release H_2_S for synergistic photothermal/H_2_S treatment.

### NIR-responsive release of H_2_S

The process of NIR-responsively generated heat to trigger the release of H_2_S from the Pd-Su nanomedicine was investigated by real-time fluorescence spectrometer monitoring. Hydrosulfide naphthalimide-2 (C_15_H_12_N_4_O_3_, HSN-2), a fluorescence probe for H_2_S, was used to monitor the release of H_2_S [37, 38]. Upon treatment with H_2_S, the HSN-2 probe will be efficiently converted to a fluorescent amine product (C_15_H_14_N_2_O_3_), thereby resulting in fluorescence turn-on. Taking the case of no NIR irradiation as blank control, the Pd-Su nanomedicine clearly showed the NIR-triggered H_2_S release behaviors under the irradiation of 808-nm laser at different power densities (Fig. 2d and Additional file 1: Figure S7). When the power density was the same, the release amount of H_2_S from the Pd-Su nanomedicine increased with the increase of NIR-irradiation time. Meanwhile, under the same irradiation time, the increase in power density resulted in more amount of H_2_S released from the Pd-Su nanomedicine. Since Pd nanoparticles can adsorb molecular oxygen and convert it into singlet oxygen under light irradiation [39, 40], we checked the possibility of singlet oxygen generation from Pd and Pd-Su. From Additional file 1: Figure S8, compared with a general photodynamic agent Ce6, the singlet oxygen generation capability of Pd was considerably weak, and that of Pd-Su was even fainter towards almost ignorable, possibly because surface Su coordination blocked the adsorption of oxygen on the surface of Pd-Su.

Encouraged by the good NIR-responsive H_2_S release performance of the Pd-Su nanomedicine in aqueous solution, we then conducted further evaluation of the H_2_S release in 4T1 cancer cells under the trigger of 808-nm laser irradiation using the WSP-5 fluorescent probe [41, 42]. Cell nuclei were stained by the 4,6-diamino-2-phenyl indole (DAPI), which shows fluorescence signal (blue) in nucleus under confocal laser light. The experiment was set up in three groups, including a blank control group, the Pd-Su nanomedicine without laser irradiation as the control group, and the Pd-Su nanomedicine with laser irradiation as the experimental group. As expected (Additional file 1: Figure S9), 4T1 cells treated with only the Pd-Su nanomedicine for 4 h showed almost no fluorescence signal (green). However, after 4T1 cells were incubated with the Pd-Su nanomedicine for 4 h and then exposed to the 808-nm laser irradiation for 10 min, strong green fluorescence can be observed in cells, indicating the intracellular NIR-responsive release of H_2_S from the Pd-Su nanomedicine under the trigger of NIR laser irradiation.

### Photothermal/H_2_S synergistic treatment in vitro

**Fig. 3:**
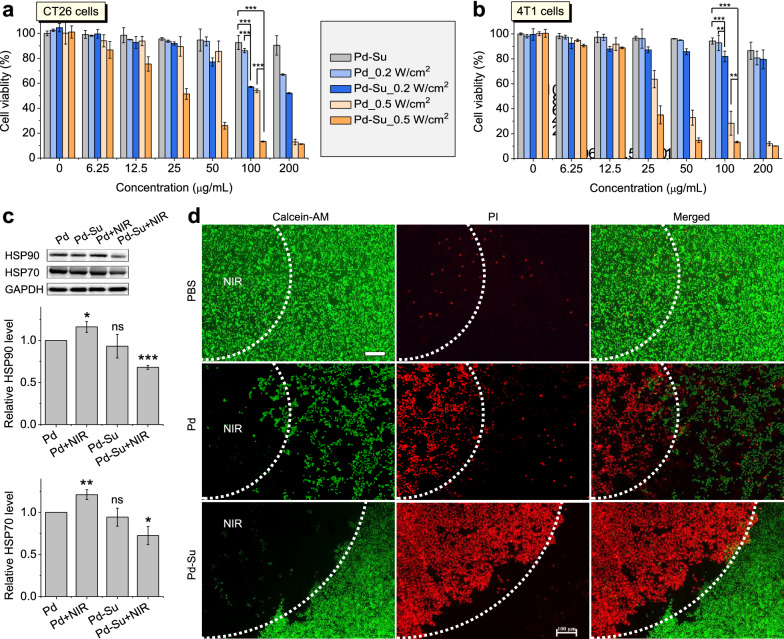
In vitro outcomes of combined photothermal/H_2_S therapy against CT26 (**a**) and 4T1 (**b**) cancer cells (*n* = 6). **c** Intracellular HSP90 and HSP70 expression in 4T1 cells with different treatments. **d** Fluorescence images of 4T1 cells co-stained with calcein-AM/PI after incubation with Pd or Pd-Su solution (200 μg mL^−1^, 4 h) and exposure to 808-nm laser irradiation (0.5 W cm^−2^, 30 min). The white dashed lines represent the boundary between the light-irradiated area and the dark area. Scale bar, 100 μm. The data were presented as mean ± SD (standard deviation). *P* values were calculated by two-tailed Student’s *t*-test (*p < 0.05; **p < 0.01; ***p < 0.001; ns, no significance)

In view of the above confirmed NIR-responsive H_2_S release and NIR-photothermal effect of the Pd-Su nanomedicine, the combination of H_2_S and hyperthermia for cancer treatment was worthy of further study in vitro. First, the cytotoxicities of Pd-Su nanomedicine and Pd nanocubes against cancer cells (4T1 and CT-26 cells) and normal cells (L02 cells) under 808-nm laser irradiation were investigated by the CCK-8 assay (Fig. 3a, b and Additional file 1: Figure S10). In the absence of 808-nm laser irradiation, the Pd-Su nanomedicine did not cause a significant decrease in cell viability. Even at a high concentration of 200 µg mL^-1^, the maximum viability decline of 4T1, CT26, and L02 cells did not exceed 9%, which demonstrated the low cytotoxic side effects of the Pd-Su nanomedicine. Under laser irradiation, both Pd-Su nanomedicine and Pd nanocubes exhibited remarkable concentration- and power density-dependent cytotoxicity against cancer cells. Notably, the Pd-Su nanomedicine induced the death of much more cancer cells than Pd nanocubes at the same particle concentration, due to the synergistic effect of photothermal heating and H_2_S. Furthermore, we found that PTT with Pd+NIR can induce the distinct up-regulation of the heat shock proteins (HSP90 and HSP70) expression in 4T1 cells, and Pd-Su+NIR sharply inhibited their expression (Fig. 3c), owing to NIR-responsive release of H_2_S from Pd-Su. It suggested that H_2_S sensitized PTT by suppressing the heat stress tolerance of cancer cells and therefore enhanced the therapeutic outcome of PTT. Moreover, the Pd-Su nanomedicine did not show obvious cytotoxicity on normal cells under the investigated concentration range (0~200 μg mL^-1^) and NIR irradiation (Additional file 1: Figure S10). This suggested that H_2_S had a protective effect on normal cells and can reduce the toxic side effects of hyperthermia on normal cells [43, 44].

To directly observe the effect of the synergistic treatment with the Pd-Su nanomedicine, 4T1 cells after various treatments were co-stained with calcein acetoxymethyl ester/propidium iodide (calcein - AM/PI) to differentiate live (green) and dead (red) cells (Fig. 3d). The white dashed line represents the boundary between the laser irradiation area and the dark area. For the cells incubated only with the culture medium, there was no obvious cell death in the illuminated area, similar to the dark area, indicating that simple laser irradiation had no special killing effect on cancer cells. After the cells were treated with Pd nanocubes, laser irradiation resulted in a significant increase in the number of dead cells, due to the killing effect of hyperthermia on cancer cells. In contrast, the Pd-Su nanomedicine plus laser stimulation caused the most serious cell death, and the cells in the irradiated area were basically killed, due to the synergistic effect of photothermal heating and H_2_S produced by the thermal hydrolysis of sulourea.

Flow cytometry was employed to assess the apoptosis rate of 4T1 cells under different treatments (Additional file 1: Figure S11). Compared with the control group, cancer cells treated with only the Pd-Su nanomedicine showed no significant increase in cell apoptosis. However, when exposed to the 808-nm laser (0.5 W cm^-2^, 8 min), the Pd-Su nanomedicine caused severe cell apoptosis (82.0%), and the apoptosis rate of this group was significantly higher than that of the Pd nanocubes + NIR laser group (75.8%). These results were in accordance with the above cytotoxicity data of CCK-8 assay and calcein-AM/PI staining.

### PAI and photothermal imaging (PTI) performance

As an emerging noninvasive imaging technology, PAI combines the advantages of good spatial resolution, high sensitivity, and deep penetration [45]. Due to the strong NIR absorption capacity and high photothermal conversion performance, the Pd-Su nanomedicine was expected to have both PAI and PTI functions for guidance of tumor treatment. To confirm this supposition, the PAI performance of the Pd-Su nanomedicine in water was evaluated by a PAI system. The full photoacoustic signal spectrum of the Pd-Su nanomedicine (λ = 680~970 nm) was first scanned, which revealed that Pd-Su had the strongest photoacoustic signal at 720 nm (Additional file 1: Figure S12). Subsequently, the PAI property of Pd-Su at 720 nm was studied in detail. As shown in Additional file 1: Figure S13a, b, it was found that the photoacoustic signal of Pd-Su nanomedicine solutions was positively correlated to its concentration within the experimental concentration range.

**Fig. 4.**
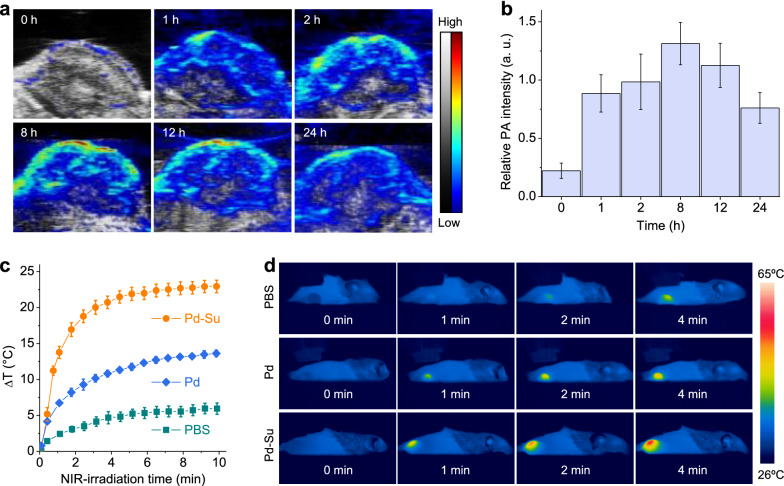
**a** PAI images of 4T1 tumor treated with the Pd-Su nanomedicine. **b** Relative photoacoustic (PA) intensity change with time after intravenous injection with the Pd-Su solution to evaluate the intratumoral accumulation and retention of Pd-Su (n = 3). The data were presented as mean ± SD. **c** Photothermal curves of tumors treated with PBS, Pd, or Pd-Su and then exposed to 808-nm laser irradiation (n = 3). **d** Photothermal images of corresponding 4T1 tumor-bearing mice at different NIR-irradiation time points.

Based on the excellent PAI performance of Pd-Su nanomedicine solutions, we conducted further evaluation on its PAI performance in vivo (Fig. 4a, b). Compared with the result of 0 h (no material was injected), the photoacoustic signal in the tumor increased rapidly at 1 h post-injection of the Pd-Su nanomedicine (10 mg kg^-1^, 100 μL), and the strongest signal was observed at 8 h. This was consistent with the analysis results from inductively coupled plasma-atomic emission spectroscopy (ICP-AES) (Additional file 1: Figure S14), which also revealed that the intratumoral accumulation of the Pd-Su nanomedicine reached the maximum at 8 h after injection (20.0% ID/g), which was equal to 40 µg Pd-Su per gram tumor for 20 g mice at an injection dose of 10 mg/kg. According to NIR-responsive H_2_S release behavior of Pd-Su in vitro (Fig. 2d), the intratumoral H_2_S level can possibly achieve about 11.5±5.8 nM after 10 min NIR irradiation at 0.5 W cm^-2^, which was close to the really measured value (13.2±6.9 nM, Additional file 1: Figure S15). Therefore, the Pd-Su nanomedicine could effectively accumulate in the tumor site via the enhanced permeability and retention (EPR) effect, in favor of tumor localization and therapy guidance. In addition, Pd-Su nanomedicine exhibited some advantages including small particle size, high dispersion, and strong coordination between Pd and Su in favor of reducing the adsorption of biomolecules on the surface. These favorable factors might reduce the possibility of Pd-Su nanomedicine being removed by the reticuloendothelial system, causing the long circulation *in vivo* after tail vein injection.

Before in vivo therapy, the NIR-photothermal effect of the Pd-Su nanomedicine in the 4T1 tumor model was monitored using a thermal imaging camera. At 4 h after tail vein injection of the Pd-Su nanomedicine (10 mg kg^-1^), tumors were irradiated by the 808-nm laser at the power density of 0.5 W cm^-2^ for 10 min. As shown in Fig. 4c, d, the phosphate buffered solution (PBS) control group showed a slight rise in temperature (only ~4 °C) at the tumor site, while the Pd-Su and Pd (10 mg kg^-1^) groups exhibited about 23 °C and 14 °C of temperature increase, respectively. It can be seen that the Pd-Su nanomedicine still exhibited much better photothermal effect than Pd nanocubes after intravenous injection. Therefore, the PTI/PAI dual-mode imaging can well guide tumor treatment.

The serum biochemistry assay and blood routine analysis were also carried out to assess the safe injection dose for in vivo cancer therapy. The results of blood biochemical analyses including liver and kidney functions were shown in Additional file 1: Figure S16, which indicated that the Pd-Su nanomedicine had almost no negative effects on the mice even at the highest injection dose of 400 mg/kg. The standard haematology markers of all tested groups (Additional file 1: Figure S17) also showed no abnormalities. Hence, the injection dose of 10 mg kg^-1^ for tumor therapy should be safe enough.

### NIR-controlled in vivo tumor treatment

**Fig. 5.**
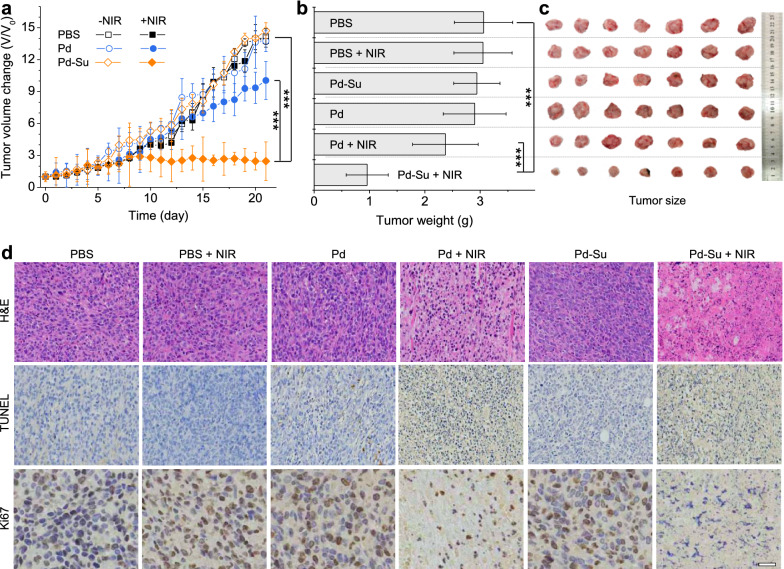
**a** Tumor growth curves of the mice treated with PBS (blank control), PBS + NIR, Pd, Pd + NIR, Pd-Su, and Pd-Su + NIR. The tumor volume was normalized to the initial tumor volume (Day 0). **b** Mean weight of the tumors harvested from the mice of various groups on the last day. The data were presented as mean ± SD. *P* values were calculated by two-tailed Student’s *t*-test (***p < 0.001; ns = no significance). **c** Photos of the tumors from various groups on the last day. **d** Histological examination of tumor sections from the mice that received various treatments by the H&E, TUNEL, and Ki 67 staining methods (scale bar, 50 μm).

Finally, in vivo evaluation of the Pd-Su nanomedicine for tumor treatment was performed using 4T1-tumor bearing mice. The mice were randomly divided into six groups, including a blank control group (PBS without NIR irradiation), four control groups (PBS + NIR, Pd, Pd + NIR, and Pd-Su), and an experimental group (Pd-Su + NIR). The mice were intravenously injected with PBS (100 μL) or the PBS solutions of the nanomaterials (10 mg kg^-1^) on days 1 and 9, and the tumors of NIR irradiation groups were exposed to 808-nm laser (0.5 W cm^-2^) for 10 min at 4 h post injection. Meanwhile, the in vivo PTI results (Fig. 4c) revealed a high temperature increase by ~23 °C (10 min NIR irradiation), which was high enough for PTT and H_2_S release. Too high injection dose will lead to vigorous photothermal response and cause the side effects of hyperthermia. Based on these considerations, we chose the injection dose of 10 mg kg^-1^ for in vivo tumor treatment. From Fig. 5a, the tumors in the blank control group (PBS without NIR irradiation) kept increasing during three weeks of monitoring. There was no significant difference in tumor growth between the blank control group and the control groups treated with PBS + NIR, Pd, or Pd-Su. The photothermal control group with Pd + NIR treatment showed tumor growth suppression of ~20.0%, owing to the photothermal effect of the Pd nanocubes. In contrast, the Pd-Su + NIR group showed remarkably higher inhibition rate of tumor growth (71.0%). The good treatment effect of the Pd-Su + NIR group confirmed the synergetic effects of photothermal heating and H_2_S produced by the NIR-responsive Pd-Su nanomedicine. The NIR laser-induced temperature increase triggered the intratumoral release of H_2_S from the Pd-Su nanomedicine (Additional file 1: Figure S15), and meanwhile, the enhanced molecular thermal movement due to photothermal heating was conducive to the penetration of H_2_S in the tumor. [15, 16, 46] These characteristics of the Pd-Su nanomedicine may be an important reason for the satisfactory synergistic therapeutic effect.

After monitoring for 21 days, all of the tumors were harvested from the treated mice for weighing (Fig. 5b), photographing (Fig. 5c), and subsequent histological analyses including hematoxylin-eosin (H&E) staining, terminal deoxynucleotidyl transferase-mediated dUTP-biotin nick end labeling (TUNEL) staining, and Ki 67 staining (Fig. 5d). The final mean tumor weight and tumor images of various groups further confirmed the best therapeutic effect of the Pd-Su nanomedicine plus laser irradiation. As indicated in the images of H&E and TUNEL staining, the tumors from the photothermal control group (Pd + NIR) exhibited a certain degree of tissue destruction and cell apoptosis, while very severe tumor damage was found in the experimental group (Pd-Su + NIR). Other control groups (PBS + NIR, Pd, and Pd-Su) showed no notable tumor destruction, similar to the blank control group. In comparison with other groups, the experimental group (Pd-Su + NIR) had the minimum expression of Ki-67 in the tumor, demonstrating that the combination of PTT and H_2_S GT can effectively inhibit the proliferation of 4T1 tumor cells.

During the whole process of treatment, the body weight of mice in different treatment groups was almost unchanged (Additional file 1: Figure S18), reflecting the high biocompatibility of Pd nanocubes and Pd-Su nanomedicine. In addition, all the mice were humanely sacrificed after 21 days of monitoring, and main organs (heart, liver, spleen, lung, and kidney) were collected for histopathological evaluation by the H&E staining. It could be found that the treatments of all groups did not bring obvious damage to main organs (Additional file 1: Figure S19), indicating no obvious systematic toxicity of the Pd-Su nanomedicine. Therefore, it can be concluded that the NIR-responsive Pd-Su nanomedicine exhibited satisfactory tumor treatment effects and low toxic side effects in vivo.

## Conclusion

In summary, the Pd-Su nanomedicine with a Su loading capacity of 85 mg/g can be conveniently prepared at room temperature by utilizing the coordination of sulourea on the surface of Pd nanocubes. Due to the surface coordination effect, the Pd-Su nanomedicine showed a significantly enhanced photothermal conversion performance, with a conversion efficiency as high as 69.4% under 808-nm laser irradiation. The temperature increase caused by the photothermal effect led to the NIR-photothermal-controlled release of H_2_S from Su, and at the same time it will aggravate the thermal movement and penetration of the surrounding H_2_S gas molecules, thereby enhancing the therapeutic effect. The in vitro and in vivo cancer treatment studies using the Pd-Su nanomedicine with these unique advantages showed that the combination of photothermal heating and H_2_S can produce a good synergetic effect to effectively inhibit tumor growth. Moreover, the good PTI/PAI dual-mode imaging performance of the Pd-Su nanomedicine can be utilized to well monitor tumor treatment. Therefore, the Pd-Su nanomedicine with good biological application prospects are worthy of further development and exploration in tumor treatment.

## Methods

### Material

Na_2_PdCl_4_ (99.9%) and ascorbic acid (99.0%) were provided by Beijing Bailing Technology Co., Ltd. PVP (M_w_=55 000 Dalton) was obtained from Sigma-Aldrich. Potassium bromide (KBr, 99.0%) was acquired from Shanghai Macklin Biochemical Co., Ltd. Su (99%) was purchased from Aladdin. All other reagents used were of the highest commercial grade available. Deionized water with a resistivity of 18.2 MΩ cm was used during the experiments.

### Synthesis of Pd nanocube

Na_2_PdCl_4_ (56.4 mg), KBr (301 mg), PVP (106.4 mg), and ascorbic acid (60 mg) were dissolved in 11 mL of deionized water. The mixture was sonicated for 2~3 min to make the reagents fully dissolved and uniformly mixed, and then the solution was kept at 80 °C for 3 h under stirring. After naturally cooling down to room temperature, the solution was centrifuged at 3000 rpm for 30 min using the Amicon hyperfiltration tubes with a molecular weight cutoff of 100 kDa to collect the Pd nanocubes. The nanocubes were washed twice with deionized water, dispersed in 10 mL of deionized water, and stored in the dark for further use.

### Synthesis of Pd-Su nanomedicine

Typically, 14.4 mg of Su was dissolved in 12 mL of deionized water, and this solution was added to the Pd nanocube suspension (10 mL, 2 mg mL^-1^) under magnetic stirring. After 3 h of stirring at room temperature, the resulting product was collected by centrifugation using the Amicon hyperfiltration tubes, washed twice, and finally dispersed in deionized water and stored in the dark.

### Characterization of the Pd and Pd-Su nanomaterials

The morphology, size, and elementary content of Pd nanocubes and Pd-Su nanomedicine were measured by an APREO high resolution SEM instrument and a JEM-F200 field emission TEM instrument with an acceleration voltage of 200 kV. The hydrodynamic size and zeta potential were measured on a Malvern Zetasizer Nano ZS90 equipped with a solid-state He-Ne laser (λ = 633 nm). The phase structures of Pd nanocubes and Pd-Su nanomedicine were characterized by powder XRD using a M21X diffractometer (Cu Kα, λ = 1.54056 Å) operated at 40 kV and 200 mA. The experimental diffraction patterns were collected with a scanning range of 10°~70° at room temperature. The FTIR absorption bands of Pd-Su nanomedicine was measured by a Thermo-Nicolet Nexus 670 FTIR spectrometer. Concentrated sample solutions were dropped on the universal diamond ATR sampling accessory and slowly air dried. UV-Vis-NIR absorption spectra of the solutions (200~1000 nm) were recorded at room temperature with an Agilent Cary 60 spectrophotometer.

### Photothermal effects of Pd nanocubes and Pd-Su nanomedicine

By monitoring the temperature change of the sample solutions irradiated by the 808-nm laser under different power densities (1.0, 0.5, and 0.2 W cm^-2^), the photothermal heating curves were obtained. The laser was provided by a fiber-coupled continuous semiconductor diode laser (KS-810F-8000, Kai Site Electronic Technology Co., Ltd), and the temperature was recorded by a fixed-mounted thermal imaging camera (FLIR A300-series). The photothermal conversion efficiencies (η) of Pd nanocubes and Pd-Su nanomedicine were calculated by Roper's reported method (Additional file 1: Equations S1 to S11).

### H_2_S release behavior of the Pd-Su nanomedicine

Pd-Su nanomedicine solutions (4 mg mL^-1^, 1 mL) were placed into 1.5-mL micro centrifuge tubes and irradiated for different time periods (0, 3, 8, 15, 20, 25, and 30 min) using an 808-nm NIR laser with a set power density (0.2, 0.5, and 1.0 W cm^-2^). At the appointed time, the solutions were immediately centrifuged at 14000 rpm for 15 min, and then the supernatant was purified by 30 min of centrifugation at 3000 rpm using the Amicon hyperfiltration tubes (MWCO = 3 kDa, Millipore) to collect the water-dissolved H_2_S. Next, 200 µL of the dissolved H_2_S was mixed with a dimethyl sulfoxide solution of H_2_S probe (0.17 mg mL^-1^, 5 µL), and diluted with deionized water to 1.5 mL. The fluorescence intensity of the mixed solutions was measured using a fluorescence spectrometer (E_x_ = 435 nm, E_m_ = 460~750 nm). To further determine the concentration of released H_2_S, Na_2_S was used as a sulfur source to react with the H_2_S probe, and a linear fitting standard curve was obtained based on the known concentrations and the corresponding fluorescence intensities at 543 nm. In addition, singlet oxygen generated from Pd and Pd-Su without or with NIR irradiation (0.5W cm^-2^, 10 min) was also measured using the 9,10-anthracenediyl-bis-(methylene)-dimalonic acid (ABDA) bleaching method.

### In vitro and in vivo detection of H_2_S released in 4T1 cells

The experiments for detection of cellular H_2_S included three groups: DMEM group (blank control, group 1), only Pd-Su group (group 2), and Pd-Su + NIR group (group 3). The 4T1 cells were seeded in laser confocal culture dishes and cultured in a humidified incubator for 12 h at 37 °C and 5% CO_2_ to ensure cell adhesion. The medium was replaced with the complete medium (1 mL, group 1) or a Pd-Su solution (200 μg mL^-1^, 1 mL, groups 2 and 3). After the cells were incubated in the incubator for another 4 h, the culture medium was carefully aspirated, and the fresh complete medium (1 mL) was added to each dish. The group 3 with an initial temperature of 37 °C was exposed to the NIR laser irradiation (808 nm, 0.5 W cm^-2^) for 10 min. Next, 4T1 cells in these three groups were incubated with the H_2_S probe WSP-5 (50 µM) for 30 min and fixed for 10 min using 4% paraformaldehyde, and then the cell nuclei were stained for 30 min with DAPI. Finally, a confocal microscope (ZEISS LSM880) was used to observe the green fluorescence signal from the H_2_S probe (E_x_ = 502 nm, E_m_ = 510~560 nm) and the blue fluorescence from the DAPI-stained nuclei (E_x_ = 364 nm, E_m_ = 430~480 nm).

For in vivo measurement, when the tumor volume reached approximately 100 mm^3^, the tumor-bearing mice were randomly divided into two groups (*n* = 3 per group): Pd-Su and Pd-Su + NIR. Pd-Su nanomedicine (10 mg kg^-1^, 100 µL) was injected into mice *via* tail vein. At 4 h post-injection, the tumors of the mice from Pd-Su + NIR group were irradiated with the 808-nm laser at 0.5 W cm^-2^ for 10 min. The tumor tissue was homogenized in lysis buffer, and then the supernatant was collected to detect H_2_S concentration using the H_2_S probe.

### CCK-8 assay to evaluate the in vitro therapy using Pd-Su nanomedicine

The mouse 4T1 breast cancer cells, CT-26 colon cancer cells, and the human L02 normal hepatocytes were purchased from Shanghai Zhong Qiao Xin Zhou Biotechnology Co., Ltd. The cells (1 × 10^4^ cells/well) planted in the 96-well plates were cultured in the DMEM medium containing 10% fetal bovine serum and 1% penicllin-streptomycin for 12 h, and then co-incubated with different concentrations of Pd-Su nanomedicine or Pd nanocubes (0~200 μg mL^-1^) for 4 h. The following treatment groups were used for comparison: only PTT groups (Pd nanocubes + NIR irradiation, 0.2 and 0.5 W cm^-2^), only material group (Pd-Su nanomedicine, without NIR irradiation), combined hyperthermia and H_2_S GT groups (Pd-Su nanomedicine + NIR irradiation, 0.2 and 0.5 W cm^-2^). After the cells were incubated with the materials, the culture medium was replaced with fresh complete medium, and the cells in the NIR laser irradiation groups were exposed to an 808-nm laser for 8 min. The cells in all groups were further incubated for 12 h, and finally the cell viability was detected by the CCK-8 method. In addition, 4T1 cells treated with nanomedicines were also collected to detect intracellular levels of HSP70 and HSP90 by standard Western blotting technique with primary antibodies directed against HSP70 (1:1000; Protein tech; catalogue number: 10654-1-AP), HSP90 (1:1000; Beyotime; catalogue number: AF1378), or glyceraldehyde 3-phosphate dehydrogenase (GAPDH; loading control; 1:5000; Protein tech; catalogue number: 10494-1-AP) and goat anti-rabbit IgG secondary antibodies linked to horseradish peroxidase (1:5000; Protein tech; catalogue number: SA00001-2). Data were analyzed by ImageJ using the GAPDH signals as an internal control.

### In vitro calcein-AM/PI fluorescence staining

The 4T1 cells were cultured in 6-well plates for 12 h, and then the culture medium was replaced with 1 mL of fresh DMEM medium, Pd nanocubes (200 µg mL^-1^, in DMEM), or Pd-Su nanomedicine (200 µg mL^-1^, in DMEM). After further culturing for 4 h, the excess materials were removed and the cells were washed three times with PBS carefully. Following the introduction of fresh medium (1 mL), each well was wrapped with the tin foil, and a small hole was opened in the tin foil for laser irradiation to form a contrast between the laser irradiation area and the dark area. The small holes were exposed to 808-nm laser irradiation (0.5 W cm^-2^) for 30 min, and then the cells were further cultured for 4 h. After the cells were co-stained with calcein-AM/PI for 30 min, a fluorescent inverted microscope (NIKON TS2) was used to take photos under the excitation of 450~490 nm (for calcein-AM) and 537.5~572.5 nm (for PI).

### In vitro flow cytometry experiments

Flow cytometry was conducted to determine the cell apoptosis. The following treatment groups of 4T1 cells were used for comparison: control group (4 h of incubation with culture medium, without NIR irradiation), only PTT group (4 h of incubation with Pd nanocubes, NIR irradiation), only material group (4 h of incubation with Pd-Su nanomedicine, without NIR irradiation), and combined PTT and H_2_S GT group (4 h of incubation with Pd-Su nanomedicine, NIR irradiation). For the only PTT group and combined therapy group, the cancer cells were exposed to an 808-nm laser at 0.5 W/cm^2^ for 8 min. After further incubation for 4 h, the cells of all groups were co-stained with the Annxin V - YF488/PI and tested by a flow cytometry (Accuri C6). The collected data were analyzed using the FlowJo V 10 software.

### In vivo PAI

Healthy female BALB/c mice (~20 g) were purchased from Guangdong Medical Experimental Animal Center. The Animal Research Management Committee of Shenzhen University approved the protocols for all animal experiments. Each mouse was injected with 4T1 cells (2×10^6^ cells/mL, 100 μL) from the hind limb to establish a 4T1 tumor-bearing mouse model.

After the mean tumor volume reached about 60 mm^3^, the mice (*n*=3 per group) were injected with Pd-Su nanomedicine (100 μL, 2 mg mL^-1^) via the tail vein. The PAI was performed on a PAI system (Vevo 2100 LAZR system, Visual-Sonic Inc.) at different time points (0, 1, 2, 8, 12, and 24 h) post injection, and the corresponding signal intensities were collected.

### Biodistribution analysis

The 4T1 tumor-bearing mice model was established for evaluating the in vivo biodistribution of Pd-Su nanomedicine. When the tumor volume reached about 120 mm^3^, the mice were injected with the PBS solution of Pd-Su (100 µL, 10 mg kg^-1^) via tail vein. The mice were randomly divided into six groups (n = 3) and dissected at 4, 8, 12, and 24 h after injection. The weight of the obtained heart, liver, spleen, lung, kidneys, and tumor of each mouse was measured. Then, these organs were digested with aqua regia, heated to dryness, and added with deionized water to a certain volume. The quantitative analysis of Pd element was performed by ICP-AES (Agilent Technologies, USA).

### In vivo PTI

After the mean tumor volume reached about 80 mm^3^, the mice were divided into three groups (3 in each group). The tumor-bearing mice were intravenously injected with PBS (100 μL, group 1, as blank control group), Pd nanocubes (100 μL, 2 mg mL^-1^, group 2), or Pd-Su nanomedicine (100 μL, 2 mg mL^-1^, group 3) via tail vein. At 4 h after injection, the tumors of the mice were irradiated with the 808-nm laser at 0.5 W cm^-2^ for 10 min. During the irradiation process, an infrared thermal imaging camera (FLIR A300-series) was used to monitor the temperature change at the tumor site.

### The liver/kidney function and hemotoxicity analyses

Healthy nude mice were randomly divided into four groups (n = 4): three Pd-Su treatment groups and the control group. The PBS solutions (100 µL) of Pd-Su (100, 200, and 400 mg kg^-1^) were intravenously injected into the mice via tail vein. The control group was received injection of only PBS. At 14th day post injection, the blood was taken from the eyes of mice to examine blood routine and blood biochemical indicators.

### In vivo tumor therapy

When the tumor volume reached 100~170 mm^3^ (designated as day 0), the treatment was initiated. The mice were randomly divided into six groups (n = 7): only PBS (group 1, as blank control group), PBS + NIR (group 2), only Pd nanocubes (10 mg kg^-1^, group 3), Pd nanocubes (10 mg kg^-1^) + NIR (group 4); only Pd-Su nanomedicine (10 mg kg^-1^, group 5), and Pd-Su nanomedicine (10 mg kg^-1^) + NIR (group 6). The mice received intravenously injection with PBS or PBS solutions of the materials (100 µL) via tail vein on days 1 and 9. The tumor areas of groups 2, 4, and 6 were irradiated by an 808-nm laser (0.5 W cm^-2^) for 10 min at 4 h post injection. The body weight and tumor volume of each mouse were recorded every day. These mice were humanely sacrificed after 21 days of monitoring, and all the tumors were collected, weighed, and then taken photos.

### H&E, TUNEL, and Ki67 staining for histological analysis

After three weeks of treatment, two 4T1 tumor-bearing mice were randomly selected from each group and humanely sacrificed. The obtained tumors were fixed in paraformaldehyde of 4% and embedded in paraffin for H&E, TUNEL, and Ki67 staining.

## Supplementary Information

**Additional file 1: Figure S1.** SEM images. **Figure S2.** TEM images. **Figure S3.** TG curves. **Figure S4.** XRD patterns. **Figure S5.** High-resolution TEM images. **Figure S6.** Photothermal conversion efficacy calculation. **Figure S7.** Fluorescence spectra of H_2_S probe. **Figure S8.** Singlet oxygen production. **Figure S9.** Cellular detection of released H_2_S. **Figure S10.** Cell viability of L02 normal cells treated with Pd-Su nanomedicine. **Figure S11.** Flow cytometry results of 4T1 cells treated with Pd-Su nanomedicine. **Figure S12.** Full photoacoustic spectrum of Pd-Su nanomedicine. **Figure S13.** Photoacoustic effects of Pd-Su nanomedicine. **Figure S14.** Biodistribution of Pd-Su nanomedicine. **Figure S15.** Intratumoral H_2_S release from Pd-Su nanomedicine. **Figure S16.** Blood biochemical analyses including liver and kidney functions. **Figure S17.** Evaluation of standard haematology markers. **Figure S18.** Body weight change curves of the mice during treatment. **Figure S19.** Histological examination by H&E staining.

## Data Availability

All data generated or analyzed during this study are included in the article and Additional file 1.
