# Supplementary material for: Sulourea-coordinated Pd nanocubes for NIR-responsive photothermal/H2S therapy of cancer
Source: J Nanobiotechnology. 2021 Oct 14;19:321. doi: 10.1186/s12951-021-01042-9 (PMC8515682; doi:10.1186/s12951-021-01042-9)
Supplement: Supplementary file 1 — Additional file 1: Figure S1. SEM images. Figure S2. TEM images. Figure S3. TG curves. Figure S4. XRD patterns. Figure S5. High-resolution TEM images. Figure S6. Photothermal conversion efficacy calculation. Figure S7. Fluorescence spectra of H2S probe. Figure S8. Singlet oxygen production. Figure S9. Cellular detection of released H2S. Figure S10. Cell viability of L02 normal cells treated with Pd-Su nanomedicine. Figure S11. Flow cytometry results of 4T1 cells treated with Pd-Su nanomedicine. Figure S12. Full photoacoustic spectrum of Pd-Su nanomedicine. Figure S13. Photoacoustic effects of Pd-Su nanomedicine. Figure S14. Biodistribution of Pd-Su nanomedicine. Figure S15. Intratumoral H2S release from Pd-Su nanomedicine. Figure S16. Blood biochemical analyses including liver and kidney functions. Figure S17. Evaluation of standard haematology markers. Figure S18. Body weight change curves of the mice during treatment. Figure S19. Histological examination by H&E staining. [file 12951_2021_1042_MOESM1_ESM.doc]

**Supporting Information**

**Sulourea-coordinated Pd nanocubes for NIR-responsive photothermal/H_2_S therapy of cancer**

*Xiaoyang Guo, Jia Liu, Lingdong Jiang, Wanjun Gong, Huixia Wu*, Qianjun He**

X.Y. Guo, Prof. H.X. Wu

The Education Ministry Key Laboratory of Resource Chemistry, Joint International Research Laboratory of Resource Chemistry of Ministry of Education, Shanghai Key Laboratory of Rare Earth Functional Materials, and Shanghai Municipal Education Committee Key Laboratory of Molecular Imaging Probes and Sensors, College of Chemistry and Materials Science

Shanghai Normal University

Shanghai 200234, P. R. China

E-mail: wuhuixia@shnu.edu.cn

Dr. J. Liu, Prof. Q.J. He

Central Laboratory, Longgang District People's Hospital of Shenzhen & The Third Affiliated Hospital (Provisional) of The Chinese University of Hong Kong, Shenzhen, Guangdong, China

E-mail: nanoflower@126.com

X.Y. Guo, Dr. L.D. Jiang, Dr. W.J. Gong, Prof. Q.J. He

Guangdong Provincial Key Laboratory of Biomedical Measurements and Ultrasound Imaging, National-Regional Key Technology Engineering Laboratory for Medical Ultrasound, School of Biomedical Engineering, Health Science Center

Shenzhen University

Shenzhen 518060, Guangdong, P. R. China

E-mail: nanoflower@126.com

*Photothermal performance of Pd and Pd-Su nanoparticles*

Photothermal heating curves of nanoparticles were plotted by monitoring the temperature change with time of the sample solutions in Eppendorf tubes under the irradiation of an 808-nm NIR laser at 0.5 W cm^-2^ (KS-810F-8000, Kai Site Electronic Technology Co., Ltd.). The temperatures were recorded by a fixed-mounted thermal imaging camera (FLIR A300-series). The photothermal conversion efficacies (η) of Pd and Pd-Su nanoparticles were calculated as follows according to Roper’s method.

Energy transfer obeys the following relationship:

$Q=Q_{NP\text{s}}+Q_{dis}-Q_{surr}=\sum_{i} m_{i}C_{p,i}\frac{dT}{dt}$ (1)

$Q_{NP\text{s}}=I(1-10^{-A_{808}})\text{η}$ (2)

$Q_{surr}=hs(T-T_{surr})$ (3)

When the system temperature increase to maximum under the 808-nm laser irradiation, the energy absorbed by the system is considered equal to the energy released to the environment. We can get the following relationship:

$Q_{NP\text{s}}+Q_{dis}=Q_{surr}=hs(T_{\max}-T_{surr})$ (4)

Taking equations (2) and (3) into equation (4), we can get the equation (5):

$\text{η}=\frac{hs\text{(}T_{\max}-T_{surr})-Q_{dis}}{I\text{(1-10}^{\text{-A}_{808}})}$ (5)

Where Q_NPs_ was measured using the aqueous solution of the nanoparticles. *Q*_dis_ was measured independently using water without the nanoparticles. *Q*_surr_ represents the heat emitted by the system to the environment. *Q* represents the energy required to increase ∆*T* of the system. *T*_max_ is the highest temperature reached after 808-nm laser irradiation. *T*_surr_ is the room temperature. *I* represents the laser power of the input. A_808_ is the absorbance of nanoparticles at 808 nm. Further, *hs* is determined based on a dimensionless driving force temperature *θ*, and *τ*_s_ is a sample system time constant.

In order to get the *hs*, we research the cooling curve. When the 808-nm laser is turned off, the system releases the heat to the environment while the system temperature gradually cooling, and we can get equation (6):

$Q_{surr}=-\sum_{i} m_{i}C_{p,i}\frac{dT}{dt}=hs(T-T_{surr})$ (6)

Assuming the system time constant is *τ*_s_

$\text{τ}_{\text{s}}=\frac{\sum_{i} m_{i}C_{p,i}}{hs}$ (7)

A dimensionless driving force temperature, hypothesis for *θ*

$\text{θ}=\frac{T-T_{surr}}{T_{\max}-T_{surr}}$ (8)

Taking equation *τ*_s_ and *θ* into equation (1) and then dealing with it, we can get the equation (9):

$\frac{\text{dθ}}{\text{dt}}=\frac{1}{\text{τ}_{\text{s}}}[\frac{Q_{NPs}+Q_{\text{d}is}}{hs(T_{\max}-T_{surr})}-\text{θ]}$ (9)

When we stop irradiating, *Q*_NPs_ + *Q*_dis_ = 0, the equation (9) is processed and get the equation (10)

$\frac{\text{dθ}}{dt}=-\frac{1}{\text{τ}_{\text{s}}}$ (10)

Integrate:

$\text{t}=-\text{τ}_{\text{s}}\text{lnθ}+\text{b}$ (11)

*τ*_s_ equals to the slope of linear equation that is obtained from simulation of linearity curve of time data (*t*) versus – ln[*θ*](http://zhidao.baidu.com/question/404094915.html?fr=iks&word=ln+theta%C8%E7%BA%CE%D4%DAoffice%D6д%F2%BF%AA&ie=gbk) during naturally cooling down to ambient temperature period. The *m*_i_ and *C*_p,i_ are the mass and heat capacities of water, respectively. Then *hs* can be obtained from equation (7) and used to calculate *η* using equation (5).

**Figure S1.** SEM images of Pd nanocubes (a) and Pd-Su nanomedicine (b). Left images: bright field (BF) mode; Right images: high angle annular dark field (HAADF) mode.

**Figure S2.** TEM images of Pd-Su nanomedicine: (a) BF mode, (b) HAADF mode. Scale: 10 nm.

**Figure S3.** TG curves of Pd nanocubes, Pd-Su nanomedicine, and Su.

**Figure S4.** XRD patterns of Pd nanocubes, Pd-Su nanomedicine, and Su.

**Figure S5.** High-resolution TEM images of Pd nanocube (a) and Pd-Su nanocube (b). Scale bar, 5 nm.

**Figure S6.** (a) Photothermal heating and cooling curves of a Pd nanocube solution (2 mg mL^-1^) under irradiation of an 808-nm laser with a power density of 0.5 W cm^-2^ which was turned off after irradiation for 5 min. (b) The plot of cooling time versus negative natural logarithm of the temperature driving force obtained from the cooling stage of Pd nanocubes.

**Figure S7.** (a) Fluorescence spectra of H_2_S probe in response to Na_2_S with different concentrations (2.5 ~ 50 μM) in dimethyl sulfoxide / H_2_O solution (λ_ex_ = 435 nm); (b) Relationship between the fluorescence intensity of the probe and H_2_S concentration.

**Figure S8.** Production of singlet oxygen from Ce6 as positive control, Pd and Pd-Su under NIR irradiation (808 nm, 0.5 W cm^-2^).

**Figure S9.** Cellular detection of released H_2_S. Confocal microscopy images of 4T1 cells co-stained with WSP-5/DAPI after incubation with Pd-Su (200 μg mL^-1^, 4 h) and subsequent treatment without/with 808-nm laser irradiation (0.5 W cm^-2^, 10 min) (WSP-5, E_x_ = 502 nm and E_m_ = 510~560 nm; DAPI, E_x_ = 364 nm and E_m_ = 430~480 nm). Scale bars, 10 µm.

**Figure S10.** Cell viability of L02 normal cells treated with Pd-Su nanomedicine (Pd-Su), Pd nanocubes plus NIR irradiation (Pd + NIR), and Pd-Su nanomedicines plus NIR irradiation (Pd-Su + NIR) (*n*=6). The data were presented as mean ± SD.

**Figure S11.** Flow cytometry results of 4T1 cells co-stained with Annexin V-YF488 / PI to determine cell apoptosis after the cells were treated with Pd-Su, Pd + NIR, or Pd-Su + NIR. Control: PBS group.

**Figure S12.** Full photoacoustic spectrum of Pd-Su nanomedicine (λ = 680 ~ 970 nm).

**Figure S13.** Photoacoustic effects of Pd-Su nanomedicine. (a) PAI images of Pd-Su solutions with different concentrations. (b) The linearly fitted standard curve of photoacoustic signal intensity versus the concentration of Pd-Su solutions.

**Figure S14.** ICP-AES data of Pd element to assess the biodistribution of Pd-Su nanomedicine at different time points (4, 8, 12, and 24 h) post injection (n = 3). The data were presented as mean ± SD.

**Figure S15.** Intratumoral H_2_S levels in 4T1 tumor-bearing mice injected with Pd-Su before and after NIR irradiation (808 nm, 10 min, 0.5 W cm^-2^). The data were presented as mean ± SD (*n* = 3). *P* values were calculated by two-tailed Student’s *t*-test (**p* < 0.05).

**Figure S16.** Blood biochemical analyses including liver and kidney functions at 14th day post injection of Pd-Su nanomedicine (n = 4). ALT (U/L), alanine transaminase; AST (U/L), aspartate transaminase; ALP (U/L), alkaline phosphatase; BUN (mM), blood urea nitrogen; Scr (µM), serum creatinine. The data were presented as mean ± SD.

**Figure S17.** Evaluation of standard haematology markers including WBC, RBC, HGB, LYM, HCT, MCV, MCHC, and RDW-SD at 14th day post injection of Pd-Su nanomedicine (n = 4). WBC (10^10^/L), white blood cells; RBC (10^11^/L), red blood cells; HGB (10 g/L), haemoglobin; LYM (%), lymphocytes percentage; HCT (%), haematocrit; MCV (%), mean corpuscular volume; MCHC (100 g/L), mean corpuscular haemoglobin concentration; RDW-SD (fL), red blood cell volume distribution width. The data were presented as mean ± SD.

**Figure S18.** Body weight change curves of the mice during different treatments for 21 days. The data were presented as mean ± SD.

**Figure S19.** Histological examination by H&E staining of the main organs (heart, liver, spleen, lung, and kidney) from the PBS, PBS + NIR, Pd, Pd + NIR, Pd-Su, and Pd-Su + NIR groups after 21 days of monitoring. Scale bars, 200 µm.
